# Improved Surgical Management of Complex Neonates With Heterotaxy Syndrome

**DOI:** 10.1177/21501351251345791

**Published:** 2025-07-11

**Authors:** Alexander C. Mills, Ashley E. Dawson, Michael C. Scott, Alexis M. Kennedy, Beau A. Bequeaith, Ioannis Zoupas, Jorge D. Salazar, Damien J. LaPar

**Affiliations:** 1 McGovern Medical School at UTHealth, Houston, TX, USA; 2 Department of Pediatric Cardiovascular Surgery, Children's Heart Institute at Children's Memorial Hermann Hospital, 12340 The University of Texas Health Science Center at Houston, Houston, TX, USA

**Keywords:** heterotaxy, neonatal, CHD, surgical outcomes, single ventricle anatomy

## Abstract

**Background:** Neonatal management of congenital heart defects (CHD) among heterotaxy patients remains challenging due to significant heterogeneity in cardiac and visceral phenotypes. This study evaluated contemporary surgical outcomes and identified high-risk anatomic substrates. **Methods:** A total of 41 heterotaxy CHD patients who underwent neonatal surgical repair and/or palliation over a 10-year period were evaluated at a single institution. Heterotaxy anatomy was characterized according to right atrial isomerism (RAI) or left atrial isomerism (LAI), and other cardiac defects. Multivariate Cox regression and Kaplan-Meier analyses evaluated operative and intermediate-term outcomes. **Results:** Median age at initial operation was 7.0 days, and median operative weight was 3.1 kg. Median follow-up was 2.3 years. Of the total 41 patients, 27 (66%) had RAI, and 14 (34%) had LAI. Functional single ventricle anatomy was present in 30/41 patients (73%); 28/30 (93.3%) patients achieved stage II bidirectional Glenn and 14/30 (46.7%) achieved stage III Fontan completion; 1/30 (2%) patient underwent biventricular conversion. Operative mortality was 9.8% (4/41) after the initial operation. Permanent pacemaker placement was more common in patients with LAI. Interstage death rate was 10%, with no deaths after Fontan. Obstructed total anomalous pulmonary venous connection (TAPVC) was a risk factor for overall mortality (hazard ratio [6.0]; 95% confidence interval, 1.2-31.2; *P* = .033). Kaplan-Meier survival analysis demonstrated decreased five-year survival in RAI patients, LAI patients with single ventricle physiology, and RAI patients with obstructed TAPVC. **Conclusions:** Cardiac surgical outcomes for neonates with heterotaxy syndrome have significantly improved. Right atrial isomerism anatomy, single ventricle LAI patients, and RAI patients with obstructed TAPVC are associated with increased operative risk.

## Introduction

Heterotaxy syndrome, characterized by right atrial isomerism (RAI) versus left atrial isomerism (LAI) is a rare disorder that contains a spectrum of extracardiac and cardiac congenital defects, which present a unique challenge for congenital heart surgeons.^1^ Intestinal complications (ie, malrotation), asplenia, impaired respiratory dysmotility, and several congenital heart lesions are some of the common malformations seen.^2,3^ Neonatal management of congenital heart defects (CHD) among heterotaxy patients remains challenging due to significant heterogeneity in cardiac and visceral phenotypes. Due to the complexity of these patients, early diagnosis and referral for appropriate multidisciplinary management is essential.^4-7^

Historically, cardiac surgical outcomes in the presence of heterotaxy syndrome reported high morbidity and mortality, with an operative mortality rate as high as 15% to 30%.^8,9^ Asplenia, the need for abdominal surgery, single ventricle physiology, low birth weight, and several other variables contribute to the complexity of management and contribute to postoperative complications.^8,10,11^ Other studies have compared CHD cohorts in terms of RAI versus LAI anatomy, with RAI patients seemingly being the higher risk substrate.^12,13^ Additionally, when evaluating the effects of single ventricle physiology in heterotaxy patients, RAI patients do much worse especially when obstructive total anomalous pulmonary venous connection (TAPVC) is also present.^14^

The purpose of this study was to evaluate whether contemporary surgical management has improved neonatal surgical outcomes for this high-risk patient population as most series are small, dated, and may not accurately reflect the impact of evolving surgical and perioperative management techniques. In addition, we sought to identify important anatomic substrates to aid in the identification of further at-risk patients. We hypothesized that there would be risk differences between the two atrial isomerisms, and the presence of other specific cardiac lesions would compound this risk.

## Material and Methods

This study was approved by the institutional review board at McGovern Medical School at The University of Texas Health Science Center at Houston and the affiliated Memorial Hermann Health System (HSC-MS-23-0983). Data handling and manuscript preparation were done in accordance with the latest iteration of the Declaration of Helsinki.^15^

### Patient Selection and Consent

We performed a retrospective review of patients with heterotaxy syndrome who underwent cardiac surgery in the neonatal period at the University of Texas Houston Children's Heart Institute/Children's Memorial Hermann Hospital. Patients were identified as having undergone surgical repair or palliation over a 10-year period (2011-2021). Inclusion criteria were any heterotaxy patient who underwent cardiac surgery as a neonate (<30 days old).

### Determination of Left Atrial Isomerism or Right Atrial Isomerism

Patient anatomy was characterized according to atrial isomerism. The primary determinant was surgical anatomy reported in the operative report based off direct inspection of atrial appendage morphology. Additional information, if needed, was obtained from echocardiographic and imaging reports. Structural characteristics more common among RAI patients included discordant ventriculoarterial connections or anomalous pulmonary venous drainage anomalies. LAI patients more commonly had interrupted inferior vena cava (IVC) anomalies or symmetric pulmonary venous connections.^16^

### Outcomes and Statistical Analysis

Primary outcomes were operative mortality and intermediate- to mid-term survival rates. Operative mortality was defined as death within 30 days of the initial neonatal operation. Descriptive characteristics and outcomes were compared between the two groups by using χ^2^ for binary variables and Mann-Whitney *U* for continuous variables. Short-term and midterm survivals were estimated using Kaplan-Meier methods, which were compared between atrial isomerisms, univentricular or biventricular function, and obstructed TAPVC. Multivariate Cox regression analyses were used to identify risk-adjusted factors associated with intermediate survival. The variables included in the multivariate analysis were selected based on findings from previously published literature. Secondary outcomes focused on postoperative events, including permanent pacemaker placement, hospital length of stay, and interstage palliation outcomes. Statistical significance was defined by a *P* value <.05. All statistical analysis was performed in RStudio (RStudio version 4.2.1).

## Results

A total of 41 neonates with heterotaxy syndrome underwent cardiac surgery during the study period. Twenty patients (48.8%) were female, and 10 (24.4%) were premature. The median birth weight was 3.0 kg (interquartile range: 2.6-3.3). Right atrial isomerism was present in 27 patients (66%), and LAI was present in 14 (34%). The median age at initial operation was 7.0 days (interquartile range: 4.0-13.0), and LAI patients underwent surgery slightly earlier when compared with RAI patients (4.5 days vs 9.0 days; *P* = .237). The median weight at initial operation was 3.1 kg (interquartile range: 2.7-3.2). The median length of stay was 49.2 days (interquartile range: 33.0-96.0). Median study follow-up was 2.3 years (interquartile range: 0.5-5.8).

### Right Atrial Isomerism Versus Left Atrial Isomerism Anatomy

Table 1 displays patient and anatomic characteristics stratified by RAI and LAI atrial anatomy. Right atrial isomerism patients were more likely to have an aberrant superior vena cava (SVC) connection (left or bilateral SVC) but a normal IVC, and LAI patients were more likely to have an aberrant IVC connection (interrupted or separate IVC) and a normal right SVC. Additionally, TAPVC was more common in RAI patients, and five of six patients with obstructed TAPVC were RAI patients. Functional single ventricle anatomy was present in 73.2% of patients (n = 30/41) and was slightly more common in RAI patients. Right atrial isomerism patients were also more likely to have a common atrioventricular valve and a discordant ventriculoarterial connection. Lastly, asplenia was more common in RAI patients.

**Table 1.** Patient Demographics and Anatomic Characteristics for Neonatal Heterotaxy Patients Stratified by Atrial Isomerism.

	RAI (N = 27)	LAI (N = 14)	Total (N = 41)	*P* value
*Demographics*				
Female	14 (51.9%)	6 (42.9%)	20 (48.8%)	.585
Premature birth	6 (22.2%)	4 (28.6%)	10 (24.4%)	.653
Birth weight (kg)	3.0 (2.6-3.2)	3.1 (2.7-3.4)	3.0 (2.6-3.3)	.770
Age at operation (days)	9.0 (4.5-14.0)	4.5 (4.0-11.5)	7.0 (4.0-13.0)	.237
Weight at operation (kg)	3.1 (2.7-3.2)	3.0 (2.7-3.5)	3.1 (2.7-3.2)	.487
Chromosomal abnormality	6 (22.2%)	4 (28.6%)	10 (24.4%)	.653
*Anatomic* *characteristics*				
Cardiac position				
Dextrocardia	14 (51.9%)	5 (35.7%)	19 (46.3%)	.326
Levocardia	12 (44.4%)	8 (57.1%)	20 (48.8%)	.441
Mesocardia	1 (3.7%)	1 (7.1%)	2 (4.9%)	.628
Systemic vein connection				
Left SVC	11 (40.7%)	2 (14.3%)	13 (31.7%)	.084
Right SVC	9 (33.3%)	7 (50.0%)	16 (39.0%)	.215
Bilateral SVC	7 (25.9%)	5 (35.7%)	12 (29.2%)	.514
Interrupted IVC with hemiazygous continuation	0 (0.0%)	2 (14.3%)	2 (4.9%)	–
Interrupted IVC with azygous continuation	0 (0.0%)	7 (50.0%)	7 (17.1%)	–
Separate IVC—hepatic vein orifice	3 (11.1%)	3 (21.4%)	6 (14.6%)	.375
Normal IVC	24 (88.9%)	2 (14.3%)	26 (63.4%)	<.001
Pulmonary vein connection				
Normal pulmonary venous drainage	7 (25.9%)	10 (71.4%)	17 (41.5%)	.005
Anomalous pulmonary venous drainage, obstructed	5 (18.5%)	1 (7.1%)	6 (14.6%)	.329
Anomalous pulmonary venous drainage, nonobstructed	15 (55.6%)	3 (21.4%)	18 (43.9%)	.037
Atrioventricular connection				
Single ventricle	20 (74.1%)	10 (71.4%)	30 (73.1%)	.856
Two ventricle	8 (29.6%)	5 (35.7%)	13 (31.7%)	.691
Atrioventricular valve morphology				
Common atrioventricular valve	16 (59.3%)	2 (14.3%)	18 (43.9%)	.006
Mitral valve	1 (3.7%)	1 (7.1%)	2 (4.9%)	.629
Tricuspid valve	4 (14.8%)	3 (21.4%)	7 (17.1%)	.594
Mitral and tricuspid valve	6 (22.2%)	8 (57.1%)	14 (34.1%)	.025
Ventriculoarterial connection				
Concordant (aorta from left ventricle)	7 (25.9%)	7 (50.0%)	14 (34.1%)	.123
Discordant (aorta from right ventricle)	20 (74.1%)	7 (50.0%)	27 (65.9%)	.123
Pulmonary outflow stenosis/atresia	19 (70.4%)	7 (50.0%)	26 (63.4%)	.199
Coronary anomaly	3 (11.1%)	2 (14.3%)	5 (12.2%)	.768
Spleen				
Asplenia	15 (55.6%)	5 (35.7%)	20 (48.8%)	.228
Polysplenia	1 (3.7%)	0 (0.0%)	1 (2.4%)	–
Normal spleen	11 (40.7%)	9 (64.3%)	20 (48.8%)	.153

Abbreviations: IVC, inferior vena cava; LAI, left atrial isomerism; RAI, right atrial isomerism; SVC, superior vena cava.

### Single Ventricle Palliation

**Figure 1.**
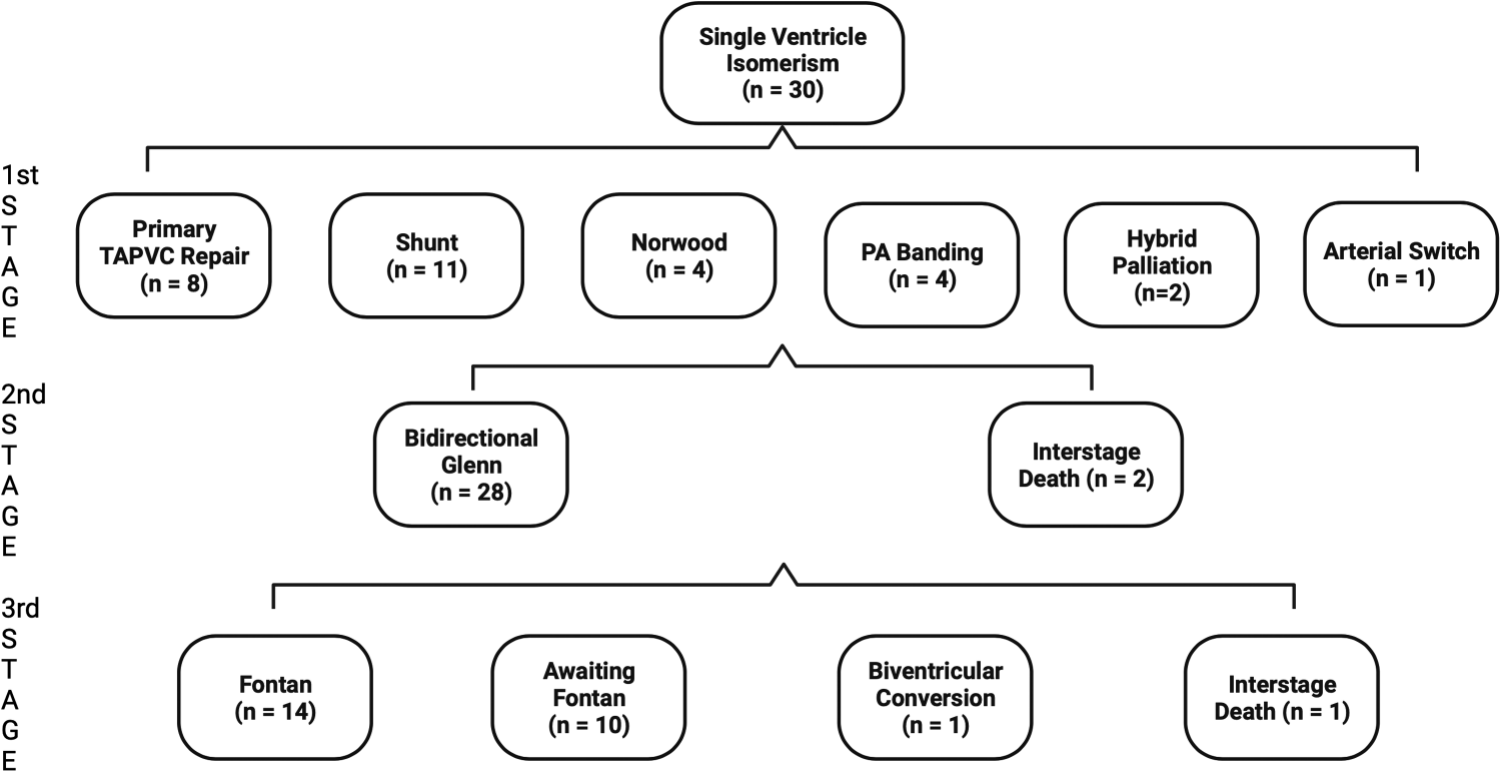
Surgical pathway for all single ventricle atrial isomerism patients. PA, pulmonary artery; TAPVC, total anomalous pulmonary venous connection.

Thirty patients (73.2%) underwent single ventricle palliation. Primary TAPVC repair occurred in eight patients. Those without primary TAPVC repair underwent isolated systemic-pulmonary artery shunting (n = 11), Norwood procedure (n = 4), PA banding (n = 4), or hybrid palliation (n = 2). A total of 28/30 patients (93.3%) achieved stage II BDG (20 RAI patients vs 8 LAI patients; *P* = .63) and 14/30 (46.7%) achieved stage III Fontan completion (10 RAI patients vs 4 LAI patients; *P* = .70). The average age at Fontan was 2.93 years (±0.59). Importantly, 1/30 (2.7%) patient underwent biventricular conversion at four years in a patient with LAI. Figure 1 depicts the surgical pathways for all atrial isomerism patients.

### Operative Outcomes

**Figure 2.**
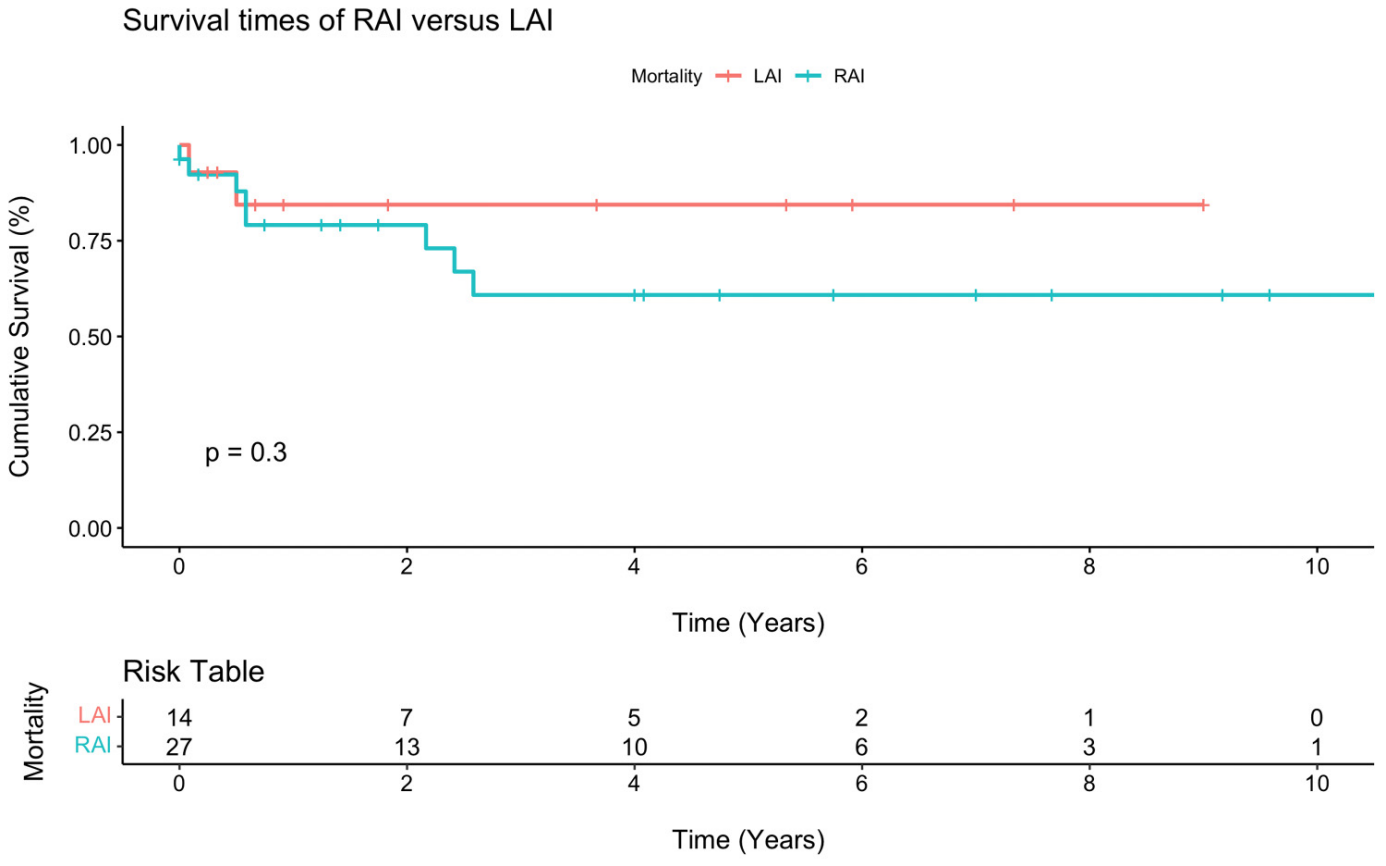
Kaplan-Meier survival curve for right atrial isomerism (RAI) versus left atrial isomerism (LAI).

**Figure 3.**
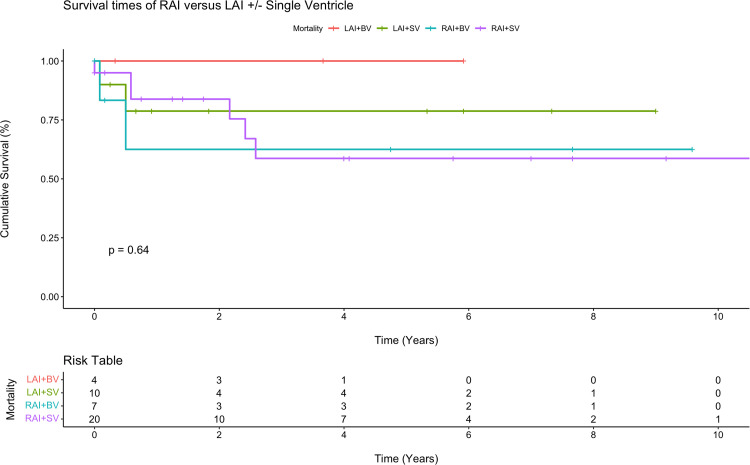
Kaplan-Meier survival curve for right atrial isomerism (RAI) versus left atrial isomerism (LAI) with single ventricle (SV) or biventricular (BV) physiology.

**Figure 4.**
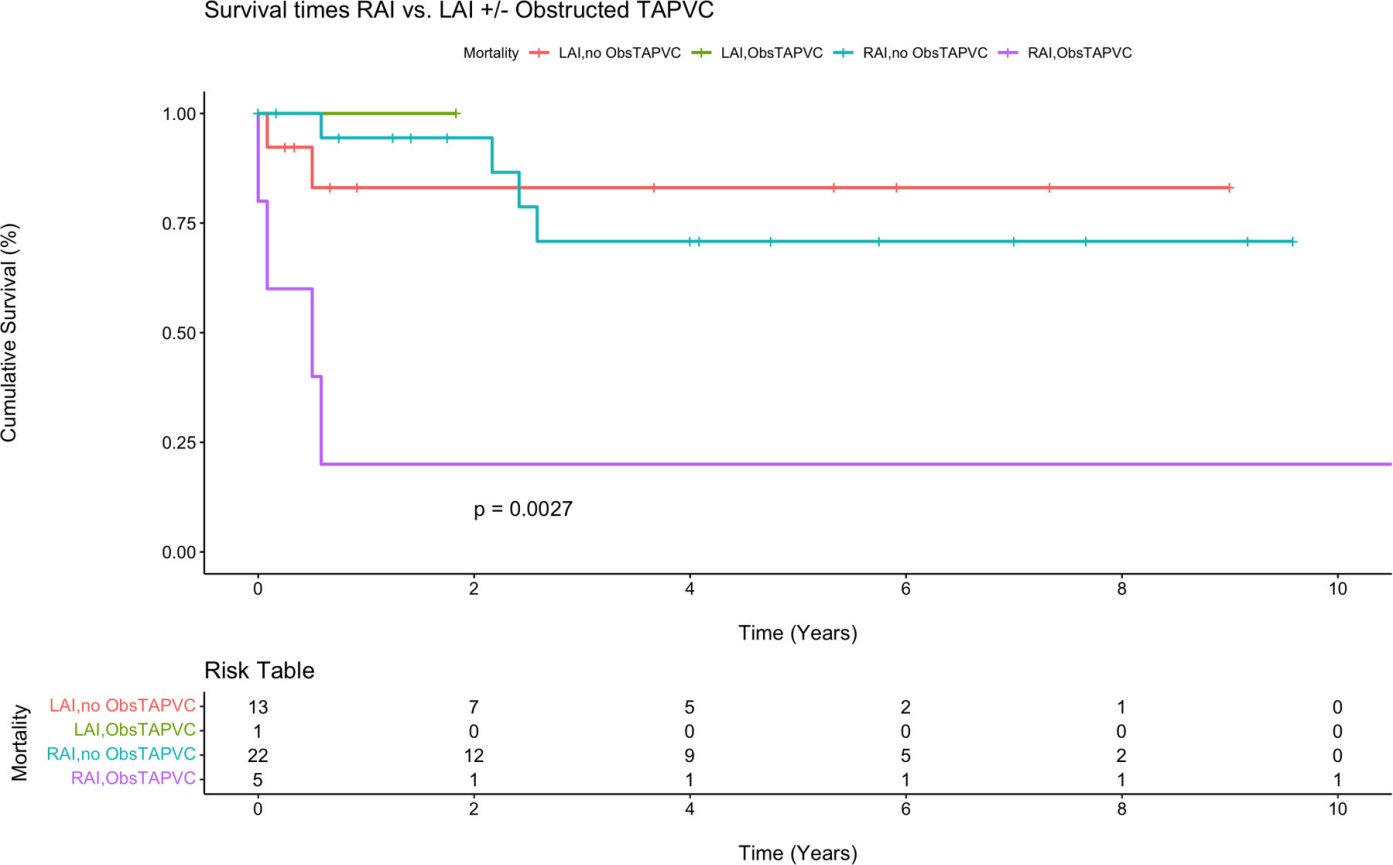
Kaplan-Meier survival curve for right atrial isomerism (RAI) versus left atrial isomerism (LAI) with obstructed total anomalous pulmonary venous connection (ObsTAPVC) or no obstructed total anomalous pulmonary venous connection biventricular (no ObsTAPVC).

Postoperative outcomes are displayed in Table 2, and survival estimates for each group are displayed in Table 3. The overall operative death rate after neonatal repair or palliation was 9.8% (n = 4/41), 14.3% (2/14) in the LAI group versus 7.4% (2/27) in the RAI group, *P* = .48. Permanent pacemaker implantation was performed in 35.7% (5/14) of LAI patients and 11.1% (3/27) of RAI patients (*P* = .13). Interstage death occurred in 3 of the 30 single ventricle patients (10.0%), including death after initial palliation in two patients (Patient 1: following pulmonary vein stenosis repair with upsizing systemic-to-pulmonary artery shunt; Patient 2: following a delayed Norwood after hybrid stage 1 palliation) and after stage 2 palliation in 1 patient (following bidirectional Glenn). There were no deaths after the Fontan operation. A multivariate Cox regression model for survival that adjusted for presence of RAI, chromosomal abnormalities, single ventricle physiology, obstructed TAPVC, asplenia, birth weight, and operative age in days demonstrated that obstructed TAPVC was a significant risk factor for reduced survival (six-fold decrease in survival) among heterotaxy patients (hazard ratio [6.0]; 95% confidence interval, 1.2-31.2; *P* = .033). Kaplan-Meier survival analysis demonstrated a five-year survival rate of 60.8% in RAI patients versus 84.4% in LAI patients (*P* = .30, Figure 2). Among anatomic subgroups, LAI patients with single ventricular physiology were associated with lower survival rates at five years when compared with biventricular physiology patients; however, there were few differences between these groups among RAI patients. No statistically significant difference was observed between any groups (Figure 3). Finally, obstructed TAPVC had a large effect among RAI patients, where five-year survival was only 20.0% (Figure 4).

**Table 2.** Operative Outcomes for All Neonatal Heterotaxy Patients Undergoing Cardiac Surgical Repair and/or Palliation.

	RAI (N = 27)	LAI (N = 14)	*P* value
*Operative outcomes*			
Operative mortality	2 (7.4%)	2 (14.3)	.482
Permanent pacemaker	3 (11.1%)	5 (35.7%)	.13
Hospital length of stay	42.8 (33.0-76.5)	79.5 (35.7-103.0)	.496

Abbreviations: LAI, left atrial isomerism; RAI, right atrial isomerism.

**Table 3.** Intermediate-Term Kaplan-Meier Survival Estimates for Several Groups of Atrial Isomerisms and Other Risk Factors.

	Survival estimates			
	1 year	3 year	5 year	*P* value
Comparison between atrial isomerisms				.3
RAI (n = 27)	79.10%	60.80%	60.80%	
LAI (n = 14)	84.40%	84.40%	84.40%	
Comparison between atrial isomerisms ± single ventricle physiology				.48
RAI, SV (n = 19)	82.90%	64.50%	64.50%	
RAI, BV (n = 8)	68.60%	51.40%	51.40%	
LAI, SV (n = 9)	76.20%	76.20%	76.20%	
LAI, BV (n = 5)	100%	100%	100%	
Comparison between atrial isomerisms ± Obstructed TAPVC				.003
RAI, obstructed TAPVC (n = 5)	20.00%	20.00%	20.00%	
RAI, no obstructed TAPVC (n = 22)	94.40%	70.80%	70.80%	
LAI, obstructed TAPVC (n = 1)	100.00%	100.00%	100.00%	
LAI, no obstructed TAPVC (n = 13)	83.10%	83.10%	83.10%	

Abbreviations: BV, biventricular; LAI, left atrial isomerism; RAI, right atrial isomerism; SV, single ventricular; TAPVC, total anomalous pulmonary venous connection.

## Comment

This retrospective review analyzed 41 neonates with heterotaxy who underwent cardiac surgical repair and/or palliation, comparing anatomic characteristics and surgical outcomes between RAI and LAI, as well as the impact of certain anatomic substrates on outcomes in this patient population. The reported results demonstrate that while RAI patients commonly presented with TAPVC, asplenia, and discordant ventriculoarterial connections, LAI patients more commonly presented with aberrant IVC connections and normal pulmonary venous drainage. LAI patients more commonly underwent permanent pacemaker implantation. Most importantly, these data demonstrate an improvement in operative mortality and intermediate survival in the modern surgical era compared with previous reports. In this analysis, we also sought to identify specific heterotaxy anatomic surgical cohorts that may be at higher risk for mortality and intermediate survival. These results identified functional single ventricular physiology (especially among LAI patients), and RAI anatomy with obstructed TAPVC as high-risk surgical cohorts for neonatal cardiac surgical repair and/or palliation. Overall, these data are critical to guide preoperative risk stratification and counseling of parents and families for this unique patient population.

The principal finding of an overall 9.8% (4/41) operative mortality rate for neonates with heterotaxy undergoing cardiac surgery in the present report compares very favorably with those reported for this patient population elsewhere. In fact, operative death rates in previously reported series are reported at 15% to 30% in patient populations that include both neonatal and non-neonatal patients.^8,9,11-13^ Thus, the improved mortality rate in our series represents a dramatic improvement from earlier studies that showed neonates with heterotaxy had a 13 times higher risk of death compared with patients undergoing initial surgery after 30 days, or that these patients had an early survival rate of less than 40%.^17,18^ Our results were in accordance with the most recent outcomes reported by a 2025 Japan-based multicenter study with 561 heterotaxy patients.^19^ The improvement in neonatal outcomes in our experience is likely multifactorial. Early detection and diagnosis play a role to facilitate appropriate transfer to tertiary care centers for multidisciplinary management.^5-7^ Increased utilization of cardiac imaging in the preoperative setting can aid the surgical team with operative planning.^20-22^ Also, surgical techniques continue to evolve and improve over time as well as perioperative care, while surgical experience plays a significant role, as there is direct volume-outcome correlation in the management of heterotaxy syndromes.^19,23^ More specifically, our institution is very aggressive at addressing TAPVC and atrioventricular valve dysfunction in functional single ventricle patients in the neonatal period, an approach proven to decrease early mortality rates.^24^ In addition, we have an aggressive approach to restriction of pulmonary blood flow in the balance of Qp:Qs for all shunted and overcirculated neonates to avoid inadequate systemic perfusion. It is important to note that all systemic-to-pulmonary conduits in this study were Blalock-Taussig-Thomas (BTT) shunts, as the ductal stenting technique was introduced at our institution at a later time. We believe these surgical approaches might have contributed to the improvement of the perioperative management for these complex neonates undergoing palliation.

The present series directly compared perioperative outcomes as well as mid- to intermediate-term survival in patients with RAI versus LAI. Overall, RAI was associated with ∼25% decrease in survival at a midterm interval of five years. Loomba et al performed a systematic review to evaluate risk factors for mortality in patients with RAI and LAI.^25^ They found that RAI had worse survival initially, but there was a survival advantage past 16 years of age in 36 years of follow-up. Anagnostopoulos et al had similar findings to our study, where RAI patients had a 15% decrease in survival at five years compared to LAI patients.^26^ Their single-center study showed slightly higher survival rates than ours, but their median age at initial operation was 2.0 months compared with ours of 7.0 days. Hirose et al demonstrated that a median age younger than 28 days at the time of surgery was associated with higher mortality in heterotaxy patients, which further underscores the significance of age in determining outcomes.^19^ Additionally, in the present series, LAI patients underwent permanent pacemaker implantation more commonly (5/14, 35.7%) than RAI patients (3/27, 11.1%), which is consistent with other studies.^26,27^Several histology and autopsy studies have shown absent or hypoplastic sinus node tissue in LAI patients, and RAI patients may have duplicated sinus nodes^.28-30^

The identification of at-risk anatomic profiles among heterotaxy neonates requiring cardiac surgical intervention is an important contribution of the present study. It has been previously established that detection of RAI versus LAI cardiac anatomy can play an important role in operative planning.^31,32^ The present results further reinforce these findings, which serves an important role in preoperative risk-stratification and counseling. Based on the reported results, informative decision-making and counseling can be performed with this knowledge as RAI patients have worse initial survival rates due to surgical complexity given their preponderance for single ventricle physiology, a common atrioventricular valve, and TAPVC, and LAI patients are at increased risk for electrophysiologic disturbances. One autopsy study found that almost all RAI patients had anomalous pulmonary venous drainage and an absent coronary sinus, and most of the LAI specimens contained some form of an interrupted IVC.^16^ This study demonstrated similar findings. Such anatomic characteristics can be seen on preoperative imaging to differentiate between atrial isomerisms and should be considered with risk stratification. Furthermore, within the characterization of atrial isomerism, patients with certain concomitant congenital heart lesions can be identified for further risk stratification. For example, the presence of TAPVC is more common in RAI patients, and obstructed TAPVC has been shown to be an independent risk factor for mortality in these patients.^19,33,34^ Given the very high association demonstrated between obstructed TAPVC and mortality, a very high index of suspicion for even subtle obstructed pulmonary venous return should exist among all TAPVC patients. These data would suggest, and we advocate for, a low threshold to repair TAPVC in the neonatal period where any question of obstruction exists. Single ventricle physiology, like other congenital cardiac lesions, expectedly confers an increased operative risk in heterotaxy patients.^8,11^ Asplenia, which is more common in RAI patients, can lead to postoperative infection and sepsis due to immunosuppression.^8^ In our study, we showed that TAPVC was uncommon in patients with LAI, and that obstructed TAPVC severely affected mortality estimates in RAI patients. For single ventricle patients, there appeared to be a larger mortality increase in LAI patients. Asplenia, while not directly analyzed, was more common in the higher risk RAI patients.

This study has select limitations. The potential for inherent selection bias should be considered in any single-institution retrospective analysis. This study focused exclusively on neonatal patients and did not analyze all heterotaxy patients undergoing cardiac surgery in the study period. Additionally, there is a risk of bias in clinical decision-making at a single center due to specialized training and operative preferences built into everyday practice at academic institutions. However, our analysis included a respectable number of patients when compared with other studies referenced in this paper, and it showed that cardiac surgery in heterotaxy patients can be performed in the neonatal period with satisfactory outcomes. To address limitations, future studies may focus upon multicenters CHD data registries developed specifically for heterotaxy patients. Such efforts may help further clarify certain high-risk patients to further analyze the impact of specific surgical techniques and surgical pathways on patient outcomes. Additionally, prospective, multicenter studies may allow for greater analytic power to more completely assess this unique patient population and offer advantages over single-institution experiences.

## Conclusions

Cardiac surgical outcomes among neonatal patients with heterotaxy syndrome continue to improve in the modern era. Important anatomic characteristics, including distinct atrial appendage morphology, can help identify select patient cohorts that may be associated with higher risk for operative mortality and reduced mid- to intermediate-term survival. In addition to atrial anatomy, the presence of functional single ventricle cardiac anatomy and obstructed TAPVC are associated with an increased likelihood of morbidity and mortality. Obstructed TAPVC may confer up to a six-fold increase in the likelihood of death. These data are important for preoperative surgical risk stratification and to inform parent counseling and operative planning in this high-risk surgical population.

## Abbreviations

CHD: congenital heart defect
IVC: inferior vena cava
LAI: left atrial isomerism
MRI: magnetic resonance imaging
RAI: right atrial isomerism
STS: Society of Thoracic Surgeons
SVC: superior vena cava
TAPVC: total anomalous pulmonary venous connection
